# Neurological Examinations of Patients Initially Diagnosed With Wild‐Type Transthyretin Amyloidosis (wtATTR)

**DOI:** 10.1111/ene.70353

**Published:** 2025-09-26

**Authors:** Jakob Stögbauer, Niklas Kämpfer, Ingrid Kindermann, Angela Zimmer, Madhuri Vennavaram, Coline Dupeyrat, Clemens Klein, Laurin Schappe, Florian Rosar, Sergiu Groppa, Ulrich Dillmann

**Affiliations:** ^1^ Department of Neurology Saarland University Medical Center Homburg Germany; ^2^ Department of Internal Medicine III (Cardiology, Angiology and Intensive Care) Saarland University Medical Center Homburg Germany; ^3^ Department of Nuclear Medicine Saarland University Medical Center Homburg Germany

**Keywords:** cardiomyopathy, CTS, electrophysiology, neuropathy, neurophysiology, wtATTR amyloidosis

## Abstract

**Background:**

Relevance of wild‐type ATTR amyloidosis (wtATTR) is increasing, due to improved therapeutic and diagnostic options. Despite the significant prevalence of neurological manifestations, there remains low awareness towards the disease, resulting in delayed diagnoses and treatment commencements. Systematic clinical and neurophysiological characterisations of large neurological collectives are lacking, as well as examination of correlations between neurological and cardiological involvement.

**Methods:**

75 patients with confirmed initial diagnosis of wtATTR amyloidosis underwent standardised clinical and extended neurophysiological examination (quantitative sensory testing, nerve conduction studies, sympathetic skin response, autonomic testing). Furthermore, cardiac involvement was quantified using laboratory and clinical scores, as well as cardiac tracer uptake in scintigraphy.

**Results:**

84% of the patients suffered from carpal tunnel syndrome (CTS), 62% with bilateral involvement. Neuropathy was present in 71%; one third showed spinal stenosis. CTS operation was performed a median of 10 years before diagnosis. Clinically, the absence of Achilles reflexes and impaired pallesthesia were particularly impressive. No correlation was found between the severity of neurological symptoms and cardiological or scintigraphic parameters.

**Conclusions:**

We were able to perform a precise clinical and neurophysiological characterisation in a large cohort of patients. We detected a predominant peripheral neuropathy pattern in a large majority of patients. However, the extent of neurological damage did not correlate with cardiac involvement. The findings may contribute to enhanced awareness among neurologists, potentially leading to earlier diagnosis and initiation of treatment.

## Graphical abstract

This article provides a comprehensive overview of the findings from neurophysiological examinations in a large cohort of patients initially diagnosed with wild‐type ATTR amyloidosis. The findings may contribute to enhanced awareness among neurologists, potentially leading to earlier diagnosis and initiation of treatment.

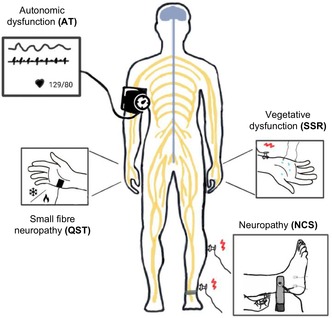

**Abbreviations**

^99m^Tc‐DPD
: Technetium‐99 m‐Diphosphono‐Propan‐Dicarbonacid
AT
: autonomic testing
BNP
: brain natriuretic peptide
CMAP
: compound muscle action potential
CTS
: carpal tunnel syndrome
e.g.: exempli gratia (lat.)
NCS
: nerve conduction studies
NYHA
: New York Heart Association
QST
: quantitative sensory testing
SNAP
: sensory nerve action potential
SSR
: sympathetic skin response
SSt
: spinal stenosis
wtATTR
: wild‐type transthyretin

## 1 Introduction

The incidence and clinical relevance of cardiac transthyretin amyloidosis (ATTR) has increased significantly in recent years, primarily due to substantial advancements in non‐invasive diagnostic methods [1], notably ^99m^Tc‐DPD bone scintigraphy [2, 3] and the emergence of novel therapeutic interventions [4, 5, 6], particularly for the wild‐type, non‐hereditary form of the disease (wtATTR). Tafamidis is approved for treatment of adults with wtATTR amyloidosis in Europe since 2021. Recent advancements in the therapeutic sector have led to the emergence of novel treatment options for ATTR cardiomyopathy. These include vutrisiran [7], which has already received regulatory approval and eplontersen [8], a drug currently undergoing clinical trials. Despite heightened awareness within the medical community, delayed diagnosis remains a significant problem for those affected. Studies have demonstrated that the time interval between initial cardiological symptoms and the diagnosis of ATTR cardiomyopathy can extend up to 4 years in many cases [9]. Consequently, the identification of extracardiac symptoms, which may signal the presence of ATTR amyloidosis and may occur prior to the cardiac manifestation, is imperative in order to achieve diagnosis as soon as possible to start early treatments [10].

The presence of amyloid fibres, which are deposited in musculoskeletal structures, is a contributing factor to the development of extracardiac symptoms [11]. This process frequently results in the onset of carpal tunnel syndrome (CTS, often manifesting bilaterally) and spinal stenosis (SSt) [12, 13, 14, 15, 16]. Additionally, ruptures in the biceps tendon and rotator cuff are commonly observed [16, 17]. Furthermore, the affection of peripheral nerve structures due to the deposition of amyloid fibres in the epineurium and its supplying blood vessels [18] can result in the development of an ATTR‐associated neuropathy [19, 20], which can also affect the small, non‐myelinated nerves (‘small fibre neuropathy’) and the autonomic nervous system [21]. The latter can lead to orthostatic dysregulation. In hereditary ATTR amyloidosis, gastrointestinal symptoms seem to be also more prevalent than reported and respond to treatment with RNA silencers [22]. Neuropathy appears to be less severe in patients with wtATTR amyloidosis than in patients with hereditary ATTR amyloidosis [23].

Given the clinical diagnoses of CTS, SSt or neuropathy, patients often present to a neurologist as their primary healthcare provider. However, it is precisely at this point that their awareness of the differential diagnosis of ATTR amyloidosis is often lacking [24]. It is therefore of utmost importance that the neurological community possesses precise knowledge of the clinical symptoms and neurophysiological constellations in affected patients in order to initiate further cardiological clarification in the event of suspicious findings. This enhanced awareness could facilitate earlier diagnosis and the initiation of treatment in patients who have previously exhibited only subclinical or minor cardiological symptoms. This could lead to an improvement in the prognosis of these patients.

Previous studies have generally been limited to examining cardiological patient collectives for the presence of extracardiac symptoms (e.g., CTS, SSt) and the time of their occurrence. However, more precise descriptions of neurological deficits by means of standardised clinical examination or even neurophysiological tests are lacking and rarely performed [24, 25]. The correlation between the severity of neurological and cardiological symptoms has also not been sufficiently investigated to date.

**FIGURE 1:**
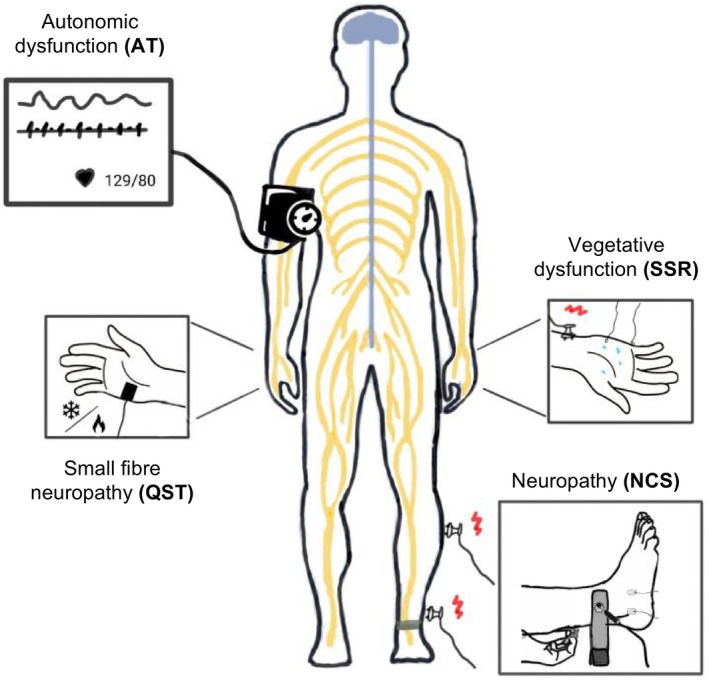
Neurophysiological features of polyneuropathy in wtATTR amyloidosis. AT, autonomic testing; NCS, nerve conduction studies; QST, quantitative sensory testing; SSR, sympathetic skin response.

The present study therefore sought to address this gap by examining patients with cardiac wtATTR amyloidosis using a standardised clinical–neurological and neurophysiological examination (electroneurography, quantitative sensory testing, sympathetic skin response, autonomic testing) at the time of initial diagnosis. Such a comprehensive neurophysiological analysis of patients with wtATTR amyloidosis has rarely been performed before. The typical neurophysiological features of polyneuropathy in wtATTR amyloidosis are reported below in Figure 1. The objective of the study is to draw conclusions about the typical neurophysiological and clinical findings of neurological patients with wtATTR amyloidosis. Furthermore, the correlation between neurological symptoms and cardiological or scintigraphic findings will be analysed.

## 2 Material and Methods

### 2.1 Patients

All patients who received a diagnosis of cardiac wtATTR amyloidosis from February 2020 to December 2024 in the Department of Internal Medicine III at Saarland University were referred to the neurophysiology department of the neurology clinic. The initial diagnosis was based on myocardial biopsy or typical scintigraphic findings.

A standardised clinical examination was conducted, and anamnestic and biographical data were documented (e.g., CTS surgery history, presence of clinically significant spinal stenosis, comorbidities, medication). The neurophysiological examination was then carried out using the methods listed below. Patients with competing underlying causes for the development of neuropathy (e.g., diabetic, ethyltoxic, B12 deficiency, chemotherapy, paraneoplastic) were identified and excluded. The cardiological and scintigraphic parameters (e.g., NYHA score, laboratory findings, Grogan score [26], Perugini score [3]) were recorded during the patient's stay in the Department of Internal Medicine III.

All patients consented to an anonymised evaluation of their data. The study protocol was approved by the local ethics committee (Ethikkommission der Ärztekammer des Saarlandes, vote No. 29/25).

### 2.2 Clinical Examination

All patients included underwent a standardised neurological examination. Cranial nerves were examined, and motor function was assessed using the Medical Research Council (MRC) Scale for Muscle Strength [27]. Reflex testing was also conducted, as well as walk and stand tests. Sensory function was assessed using manual touch, vibration, 2‐point discrimination and sense of position.

### 2.3 Neurophysiological Testing

All patients underwent nerve conduction studies (NCS) of the peripheral nerves of the upper and lower extremities. Motor NCS were performed using an external handheld stimulator with fixed cathode and anode and surface recording electrodes [28]. The sensory NCS procedure was executed utilising the orthodromic technique, employing surface electrodes for stimulation and recording. Compound motor action potential (CMAP), F‐waves, sensory nerve action potential (SNAP), motor and sensory nerve conduction velocity and distal motor latency were recorded. An analysis of the median nerve, ulnar nerve, tibial nerve, peroneal nerve and sural nerve was conducted to ascertain the presence of any potential abnormalities. Reference values were determined on the basis of internally established standard collectives, and these can be found in the results section.

Small fibre/C‐fibre involvement was measured by quantitative sensory testing (QST) as a psychophysical method. For this purpose, reaction to thermal exposure was assessed using the ‘limits method’, in accordance with previously described procedures [29]. To avoid bias, four repetitions per temperature quality were performed for each patient. Values were taken from an in‐house standard collective serving as a control group.

Autonomic function was tested using the supine‐to‐stand test (also psychophysical), Valsalva manoeuvre and respiratory sinus arrhythmia, as measured in the deep breathing test [30]. Due to a lack of validation, only the supine‐to‐stand test was performed in patients over 65 years of age.

Finally, vegetative dysfunction was quantified using sympathetic skin response (SSR) of the upper and lower extremities. The differential electrode was positioned on the palm or plantar surface, while the indifferent electrode was placed on the dorsal surface of the hand or foot. Standardised values were taken from German recommendations [31].

### 2.4 Statistical Analysis

Statistical analysis was performed using SPSS statistics (IBM, version 29.0.2.0).

Descriptive statistics were described by median, interquartile range and minimum/maximum or mean and standard deviation. The Shapiro–Wilk test was used to test for normal distribution. The Mann–Whitney U and Kruskal–Wallis tests were used to compare unpaired non‐parametric data; Spearman's correlation coefficient was used for correlations. Correlations were considered significant if the *p* value was < 0.05.

## 3 Results

### 3.1 Demographics, Clinical Scores and Laboratory Findings

A total of 75 patients diagnosed with wtATTR amyloidosis were included in this study, 10 of whom were female. The median age at presentation was 81 years. The time between initial diagnosis and presentation to the neurology department was approximately 2 months. Patients with a time interval longer than one year were not included. 62 of the included patients had CTS, 46 of them bilaterally. The median age at first surgery for CTS was 64.75 years; the elapsed time from surgery to diagnosis of wtATTR amyloidosis was 10 years. Neuropathy was present in 53 patients, 8 of whom had isolated small fibre neuropathy. Clinically significant spinal stenosis was found in approximately 30% of patients.

Clinical and laboratory parameters were recorded as part of the cardiological examination. More than half of the patients were classified as NYHA II. According to the amyloidosis‐specific Grogan score, 40 patients were categorised in stage 1.

^99m^Tc‐DPD bone scintigraphy data and results were available for 50 of the enrolled patients. The majority of patients had myocardial tracer uptake intensity corresponding to a Perugini score of 3. Patients with a Perugini score of 1 were diagnosed via cardiac biopsy.

At presentation, 65 patients were treatment‐naive; only 10 of the included patients had previously received tafamidis.

**FIGURE 2:**
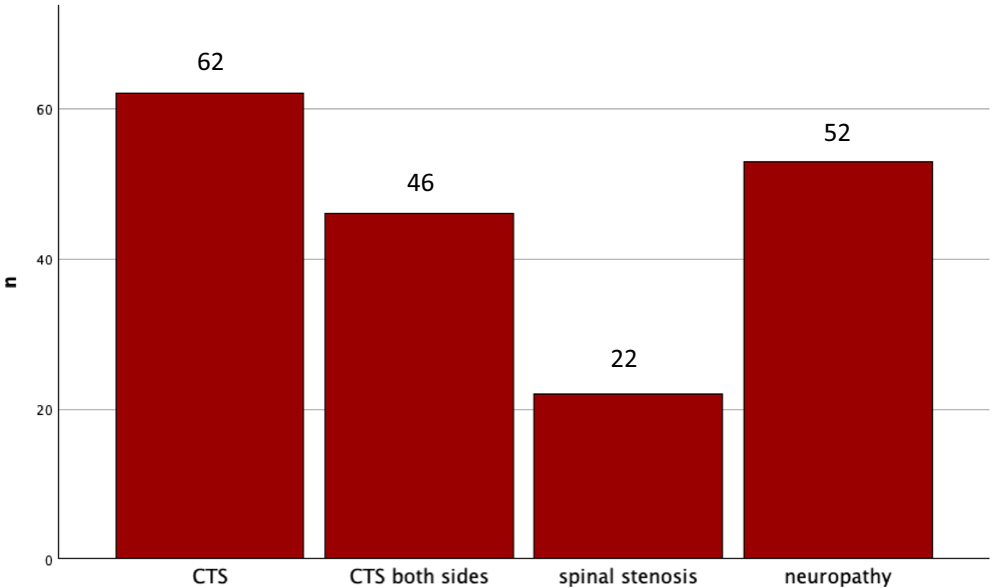
Neurological manifestations in the cohort. CTS, carpal tunnel syndrome, *n* = number of patients.

Table 1 shows demographics, clinical scores and laboratory findings of the cohort. Neurological manifestations are displayed in Figure 2.

**TABLE 1:** Demographics, clinical scores and laboratory findings.

*N*	75
Female; *n* (%)	10 (13.3)
Age; years (median; range)	81 (50–88)
Age at diagnosis; years (median; range)	80 (49–88)
Time interval between diagnosis and examination; months (median; interquartile range)	2 (5)
CTS; *n* (%)	
No	12 (16.2)
Yes	62 (83.8)
CTS both sides; *n* (%)	
No	28 (37.8)
Yes	46 (62.2)
Age at first CTS op; years (mean ± SD)	64.75 ± 10.12
Time interval between first CTS op and diagnosis; years (median; range)	10 (0–41)
Neuropathy; *n* (%)	
No	22 (29)
Yes	53 (71)
Spinal stenosis; *n* (%)	
No	53 (70.7)
Yes	22 (29.3)
NYHA; *n* (%)	
I	10 (13.9)
II	44 (61.1)
III	18 (25)
IV	0 (0)
Troponin T; pg/ml (median; range)	42 (7–202)
NT‐proBNP; mg/dl (median; range)	2008 (39–14,945)
Grogan score; *n* (%)	
1	40 (53.3)
2	20 (26.7)
3	15 (20)
Perugini score; *n* (%)	
1	5 (10)
2	13 (26)
3	32 (64)
Tafamidis therapy; *n* (%)	
No	65 (86.7)
Yes	10 (13.3)

Abbreviations: CTS, carpal tunnel syndrome; NYHA, New York Heart Association.

### 3.2 Clinical Findings

74 of the 75 patients included underwent a standardised clinical examination by an experienced neurologist. The most common clinical symptom was an extinguished Achilles reflex, followed by pathological pallesthesia at the metatarsophalangeal joint or medial malleolus and paraesthesia. Signs of paralysis were present in one third of the patients and muscle atrophy in 12 of those included.

**FIGURE 3:**
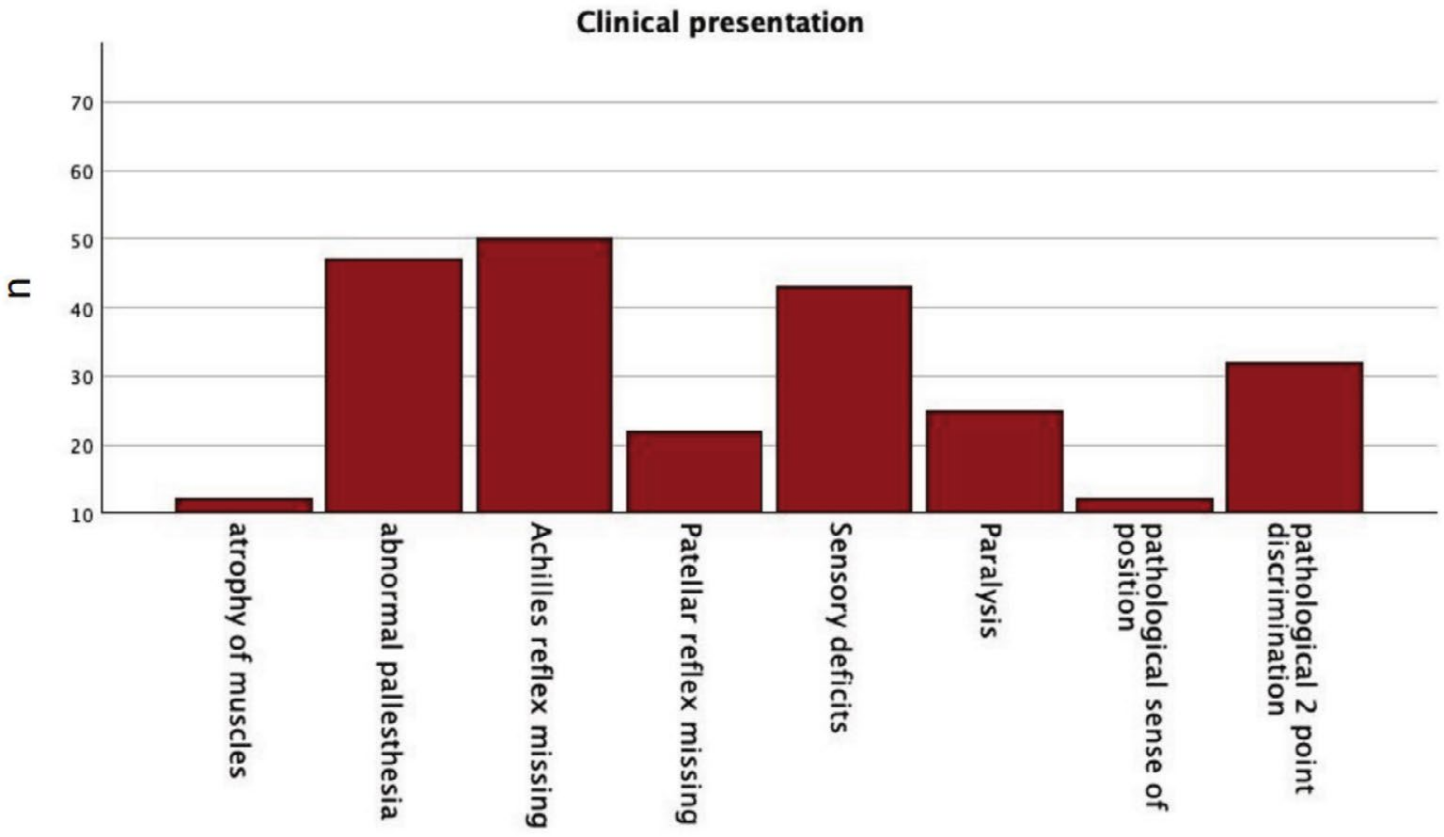
Clinical presentation. *N* = number of patients.

Clinical findings are displayed below in Table 2 and Figure 3.

**TABLE 2:** Clinical findings.

Sensory deficits; *n* (%)	
No	31 (41.9)
Yes	43 (58.1)
Atrophy of muscles; *n* (%)	
No	62 (83.8)
Yes	12 (16.2)
Pallesthesia; *n* (%)	
Normal	27 (36.5)
Pathological	47 (63.5)
Paralysis; *n* (%)	
No	49 (66.2)
Yes	25 (33.8)
Sense of position; *n* (%)	
Normal	62 (83.8)
Pathological	12 (16.2)
2 point discrimination; *n* (%)	
Normal	41 (56.2)
Pathological	32 (43.8)
Achilles reflex; *n* (%)	
Normal	24 (32.4)
Missing	50 (67.6)
Patellar reflex; *n* (%)	
Normal	52 (70.3)
Missing	22 (29.7)

### 3.3 Neurophysiological Findings

All enrolled patients underwent NCS. Due to individual limitations (technical feasibility, ability to cooperate on the part of the patient, discontinuation of examinations by the patient) quantitative sensory testing (QST) was performed in only 69 patients, autonomic testing in 58 patients and sympathetic skin response in 72 patients. Results of the testing are shown in Table 3 and Table 4.

**TABLE 3:** Neurophysiological examination—overview.

Neuropathy as measured in electroneurography; *n* (%)	
No	30 (40)
Yes	45 (60)
QST‐thermal thresholds: all in all; *n* (%)	
Normal	16 (23.2)
Pathological	53 (76.8)
QST‐thermal thresholds: hands; *n* (%)	
Normal	51 (73.9)
Pathological	18 (26.1)
QST‐thermal thresholds: feet; *n* (%)	
Normal	16 (23.2)
Pathological	53 (76.8)
Autonomous testing; *n* (%)	
Normal	27 (46.6)
Pathological	31 (53.4)
Sympathetic skin response: all in all; *n* (%)	
Normal	26 (36.1)
Pathological	46 (63.9)
Sympathetic skin response: hands; *n* (%)	
Normal	40 (55.6)
Pathological	32 (44.4)
Sympathetic skin response: feet; *n* (%)	
Normal	35 (48.6)
Pathological	37 (51.4)

**TABLE 4:** Nerve conduction studies, quantitative values.

CMAP right peroneus nerve (mV)	
*n*	62
Median (range) [Ref. > 2.5 mV]	2.29 (0–11.28)
CMAP left peroneus nerve (mV)	
*n*	33
Median (range) [Ref. > 2.5 mV]	1.96 (0–6.16)
Motor velocity right peroneus nerve (m/s)	
*n*	62
Median (range) [Ref. < 70 years: > 39 m/s; > 70 years > 35 m/s]	39 (0–48)
Motor velocity left peroneus nerve (m/s)	
*n*	33
Median (range) [Ref. < 70 years: > 39 m/s; > 70 years > 35 m/s]	38 (0–52)
SNAP right peroneus nerve (μV)	
*n*	64
Median (range) [Ref. > 2.5 μV]	1.23 (0–9)
SNAP left peroneus nerve (μV)	
*n*	35
Median (range) [Ref. > 2.5 μV]	1.18 (0–7.07)
Sensory velocity right peroneus nerve (m/s)	
*n*	64
Median (range) [Ref. < 70 years: > 39 m/s; > 70 years > 35 m/s]	39 (0–63)
Sensory velocity left peroneus nerve (m/s)	
*n*	36
Median (range) [Ref. < 70 years: > 39 m/s; > 70 years > 35 m/s]	36.5 (0–49)
CMAP right tibialis nerve (mV)	
*n*	62
Median (range) [Ref. > 2.5 mV]	2.83 (0–9.44)
CMAP left tibialis nerve (mV)	
*n*	37
Median (range) [Ref. > 2.5 mV]	2.62 (0–10.02)
Motor velocity right tibialis nerve (m/s)	
*n*	62
Median (range) [Ref. < 70 years: > 37 m/s; > 70 years > 35 m/s]	40 (0–48)
Motor velocity left tibialis nerve (m/s)	
*n*	37
Median (range) [Ref. < 70 years: > 37 m/s; > 70 years > 35 m/s]	39 (0–48)
SNAP right suralis nerve (μV)	
*n*	62
Median (range) [Ref. > 2.5 μV]	3.04 (0–26.76)
SNAP left suralis nerve (μV)	
*n*	31
Median (range) [Ref. > 2.5 μV]	1.54 (0–8.59)
Sensory velocity right suralis nerve (m/s)	
*n*	62
Median (range) [Ref. < 70 years: > 38 m/s; > 70 years > 37 m/s]	42 (0–64)
Sensory velocity left suralis nerve (m/s)	
*n*	31
Median (range) [Ref. < 70 years: > 38 m/s; > 70 years > 37 m/s]	40 (0–54)

Abbreviations: CMAP, compound motor action potential; SNAP, sensory nerve action potential.

Pathological nerve conduction studies were performed in 60% of the patients studied. QST as a measure of the few myelinated nerve fibres was abnormal in 53 patients and normal in only 16. In more than half of the patients, autonomic nervous system involvement was present, and more than 60% had a disturbed sympathetic skin response as a sign of vegetative dysregulation.

### 3.4 Association Between Neurological, Cardiological and Scintigraphic Findings

No statistically significant correlation between the presence of neuropathy, SSt or CTS and the levels of cardiac biomarkers (NT‐proBNP, troponin T) could be demonstrated in the present patient population. The same was true for the presence of pathological findings on neurography, QST, autonomic testing or SSR. The clinical severity of heart failure (NYHA score) or wtATTR amyloidosis (Grogan score) did not correlate with the absolute values obtained in the neurophysiological tests. These values did not correlate with levels of NT‐proBNP or troponin T.

The same applied to the intensity of myocardial deposition on ^99m^Tc‐DPD bone scintigraphy assessed by the Perugini score.

## 4 Discussion

This study characterised a large cohort of neurological patients with wtATTR amyloidosis in detail using real‐world data, with comparable studies only existing sporadically and comprising a smaller number of patients [25, 32, 33]. The present study's detailed clinical and neurophysiological analysis has not been commonly reported. To the best of the authors' knowledge, most (cardiological) studies have been limited to querying neurological secondary diagnoses [17]. The median time interval of only 2 months between initial diagnosis and examination allows for precise representation of the typical presentation of a neurological patient at this point in time. This short time interval is particularly noteworthy considering the known diagnostic delays in polyneuropathy, especially in wtATTR amyloidosis [34]. A plethora of studies have previously demonstrated that the screening for neurological deficits at the time of initial diagnosis is therapeutically relevant, owing to the frequently unrecognised neurological involvement. This finding was confirmed in the present study [35].

A total of 80% of the patients included in the study had CTS or a history of CTS surgery, at the time of inclusion. Furthermore, more than 60% of these patients exhibited bilateral involvement. Peripheral polyneuropathy or small fibre neuropathy was detected in more than every second patient, and clinically apparent CTS was observed in every third patient. These findings are consistent with those of previously published studies including large cohorts and emphasise the importance of neurological deficits in patients with wtATTR amyloidosis [36]. As previously reported, the present study also demonstrated that neurological impairment precedes cardiological symptoms, which ultimately usually lead to the diagnosis. In our case study, the mean interval between the initial CTS surgery and the cardiological diagnosis was 10 years. The first CTS surgery was seen as the start of the disease. Without more precise data, we can guess that the disease started even earlier. This represents a significant period during which patients were deprived of potentially effective therapy for amyloidosis due to a lack of awareness regarding the underlying disease.

The standardised clinical examination revealed symptoms consistent with neuropathy or CTS, including sensory disturbances, pathological pallesthesia and absent reflexes in the distal lower extremity. The initial presentation of this condition is non‐specific, underscoring the significance of the neurophysiological examination. The emphasis should be placed on QST, as it has been shown to be more sensitive than electroneurography. This approach facilitates the identification of any subclinical impairments in patients that may be attributable to damage to the small fibres. SSR has also been demonstrated to be of considerable assistance in this regard [37]. Research indicates that small fibre neuropathy may also be a contributing factor to gastrointestinal symptoms in patients. This is based on evidence from the hereditary form of the disease [22]. In conclusion, the combination of pathological nerve conduct studies, QST, autonomic testing and SSR is highly suggestive of the presence of wtATTR amyloidosis, particularly in cases where CTS or SSt have already been diagnosed.

The present study was unable to demonstrate a correlation between neurological involvement and the extent of cardiological damage. This result suggests that conclusions about the severity of cardiomyopathy cannot necessarily be drawn from the extent of the neurological damage. The same applies to the correlation with the cardiac tracer uptake detected by bone scintigraphy. The latter fits in with the fact that the cardiological prognosis does not seem to correlate with the Perugini grade either [38]. Analyses in this regard are not yet available in the literature and require validation by further studies with large cohorts. The greatest weakness of the present study is the assessment of the collective at a single point in time, whereby the focus should be placed on the neurological presentation at initial diagnosis. Data on the course of the disease is therefore lacking, as are any effects of specific therapy. Future observations of the course of the disease could help here. In addition, only the presence of clinically significant SSt was screened for; spinal imaging was not performed systematically. Therefore, a high number of unreported cases can be assumed. Furthermore, the QST is a patient‐dependent examination, which means that biases cannot be ruled out. One main limitation is further the high median age of the cohort. This is a key consideration in all studies dealing with wtATTR amyloidosis, given the high prevalence of the disease in the elderly population. Standard values for neurophysiological examinations are generally validated for patients under 70 years of age, so senile axonal injury may have contributed to the development of polyneuropathy. This may result in an overestimation of the prevalence of polyneuropathy in wtATTR cohorts. Further case–control studies are required in this area.

In summary, we were able to demonstrate typical biographical, clinical and neurophysiological findings in neurological patients with wtATTR amyloidosis. To the best of our knowledge, a comprehensive, holistic and detailed assessment of such a large patient cohort has never been carried out before. In particular, knowledge of the combination of these parameters can lead to earlier diagnosis by the neurologist and consecutive initiation of therapy. It was also shown that the extent of neurological damage does not necessarily correlate with cardiac impairment.

## Author Contributions

**Jakob Stögbauer:** conceptualization, investigation, writing – original draft, methodology, data curation, software, project administration, visualization, formal analysis. **Niklas Kämpfer:** conceptualization, investigation, writing – review and editing, methodology, project administration. **Ingrid Kindermann:** conceptualization, writing – review and editing, methodology. **Angela Zimmer:** conceptualization, writing – review and editing. **Madhuri Vennavaram:** investigation, writing – review and editing. **Coline Dupeyrat:** investigation, writing – review and editing. **Clemens Klein:** investigation, writing – review and editing. **Laurin Schappe:** writing – review and editing, investigation. **Florian Rosar:** investigation, writing – review and editing, methodology. **Sergiu Groppa:** validation, supervision, resources, writing – review and editing. **Ulrich Dillmann:** validation, supervision, resources, writing – review and editing, methodology, conceptualization, investigation, visualization.

## Disclosure

The authors declare that they have no conflicts of financial or non‐financial interest.

## Ethics Statement

All procedures performed in this study involving human participants were in accordance with the ethical standards of the institutional research committee and with the 1964 Helsinki Declaration and its later amendments or comparable ethical standards. All patients gave their written consent to participate in the present study. The study was approved by the local ethics committee (Ethics Committee of the Saarland Medical Association, identification number 29/25).

## Conflicts of Interest

The authors declare no conflicts of interest.

## Data Availability

Data can be provided by the authors, if requested. All figures have associated raw data. The data that supports the findings of this study is available from the corresponding author upon reasonable request.
